# Concomitant diagnosis of asthma and COPD: a quantitative study in UK primary care

**DOI:** 10.3399/bjgp18X699389

**Published:** 2018-09-25

**Authors:** Francis Nissen, Daniel R Morales, Hana Mullerova, Liam Smeeth, Ian J Douglas, Jennifer K Quint

**Affiliations:** Department of Non-communicable Disease Epidemiology, London School of Hygiene and Tropical Medicine, London.; Division of Population Health Sciences, University of Dundee, Dundee.; Real World Data and Epidemiology, GlaxoSmithKline Research and Development, Uxbridge.; Department of Non-communicable Disease Epidemiology, London School of Hygiene and Tropical Medicine, London.; Department of Non-communicable Disease Epidemiology, London School of Hygiene and Tropical Medicine, London.; National Heart and Lung Institute, Imperial College, London.

**Keywords:** asthma, COPD, electronic health records, epidemiology, primary health care, validation studies

## Abstract

**Background:**

Asthma and chronic obstructive pulmonary disease (COPD) share many characteristics and symptoms, and the differential diagnosis between the two diseases can be difficult in primary care. This study explored potential overlap between both diseases in a primary care environment.

**Aim:**

To quantify how commonly patients with COPD have a concomitant diagnosis of asthma, and how commonly patients with asthma have a concomitant diagnosis of COPD in UK primary care. Additionally, the study aimed to determine the extent of possible misdiagnosis and missed opportunities for diagnosis.

**Design and setting:**

Patients with validated asthma and patients with validated COPD in primary care were identified from the UK Clinical Practice Research Datalink (CPRD) in separate validation studies, and the diseases were confirmed by review of GP questionnaires.

**Method:**

The prevalence of concurrent asthma and COPD in validated cases of either disease was examined based on CPRD coding, GP questionnaires, and requested additional information.

**Results:**

In total, 400 patients with COPD and 351 patients with asthma in primary care were identified. Of the patients with validated asthma, 15% (*n* = 52) had previously received a diagnostic COPD Read code, although COPD was only likely in 14.8% (95% confidence interval [CI] = 11.3 to 19.0) of patients with validated asthma. More than half (52.5%, *n* = 210) of patients with validated COPD had previously received a diagnostic asthma Read code. However, when considering additional evidence to support a diagnosis of asthma, concurrent asthma was only likely in 14.5% (95% CI = 11.2 to 18.3) of patients with validated COPD.

**Conclusion:**

A concurrent asthma and COPD diagnosis appears to affect a relative minority of patients with COPD (14.5%) or asthma (14.8%). Asthma diagnosis may be over-recorded in people with COPD.

## INTRODUCTION

Worldwide, 358 million people are estimated to be affected by asthma^1^ and 174 million by chronic obstructive pulmonary disease (COPD).^2^ Both diseases can vary greatly in their presentation, and imprecision of diagnosis in both diseases remains a problem.^3^^,^^4^

Accurate diagnosis of asthma and COPD is essential as correct treatment of asthma and COPD can reduce the frequency and severity of exacerbations, and improve overall quality of life.^2^ In addition, information on chronic respiratory disease can help patients to quit smoking.

The differential diagnosis of COPD and asthma rests on differences in clinical presentation, triggering factors, and on demonstration of airflow obstruction. This airflow obstruction is not fully reversible in COPD, whereas it is in asthma. However, differential diagnosis remains difficult; the existence of asthma–COPD overlap syndrome (ACOS) remains controversial,^5^^,^^6^ as consensus regarding the clinical definition has not yet been reached. Some guidelines classify asthma cases with a persistent airway obstruction as COPD, and the two diseases are often mutually exclusive in studies to obtain unblended populations of patients with asthma and patients with COPD. In addition, the prevalence of a concomitant diagnosis of asthma and COPD varies greatly in different studies.

This study aimed to quantify the point prevalence of concomitant asthma and COPD in the diagnosed populations of both patients with asthma and patients with COPD in the UK using electronic health record databases, where validated definitions exist for the identification of both diseases. In addition, possible misdiagnosis and missed diagnosis in patients with obstructive lung diseases were examined.

## METHOD

### Study population and validation studies

**Figure 1.**
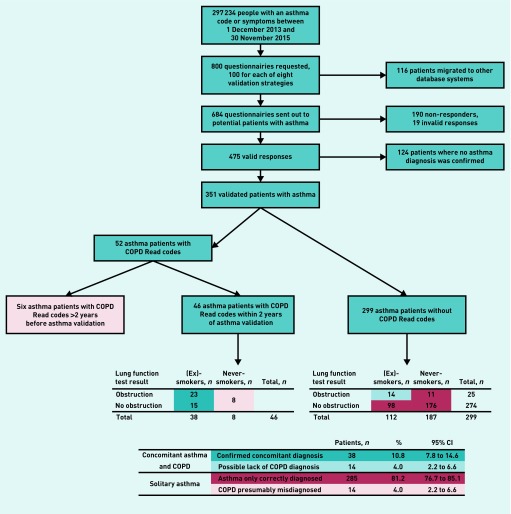
***Selection process of patients with validated asthma. COPD = chronic obstructive pulmonary disease.***

**Figure 2.**
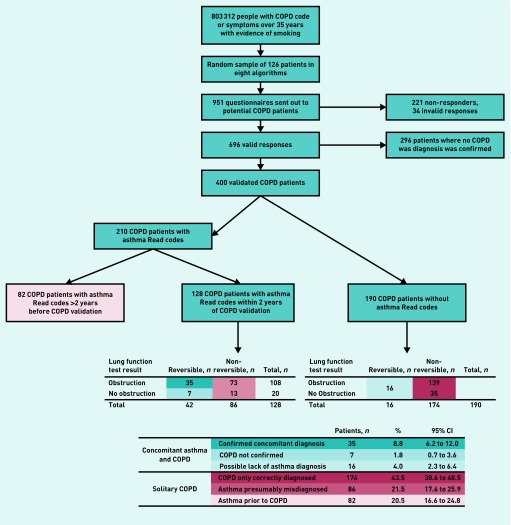
***Selection process of patients with validated COPD. COPD = chronic obstructive pulmonary disease.***

The study populations consisted of people who were included in earlier validation studies^7^^,^^8^ and are summarised in Figures 1 and 2. Questionnaires were sent out to the GPs of possible patients with asthma and patients with COPD with the intent to validate the recording of asthma and COPD in the Clinical Practice Research Datalink (CPRD). The full selection criteria of both validation studies can be found in their respective articles.^7^^,^^8^ Data collection for the asthma validation study was from 1 December 2013 to 30 November 2015. Data collection for the COPD validation study was between 1 January 2004 and 31 December 2012. In the asthma validation study, full data were only available for the patients for whom the GP stated a current asthma diagnosis and only current asthma diagnoses were considered. In the COPD validation study, the population was preselected as current or ex-smokers. The two patient populations included in this study have been thoroughly validated in their respective validation studies using these detailed GP questionnaires and requested supporting information, including outpatient referral letters, other emergency department discharge letters, airflow measurements, and radiography records. In the validation studies, the positive predictive value was 86.5% (range 77.5–92.3%) for COPD^8^ and 86.4% (range 77.4–95.4%) for asthma^7^ when only using a single diagnostic code for the respective disease.

**How this fits in**

The prevalence of concomitant asthma and COPD is likely to be overestimated in studies using only electronic health records as their symptoms are similar. This study reports on this issue by including only patients with validated asthma and patients with validated COPD from two previous validation studies. It was found that concurrent asthma and COPD diagnosis affected a relative minority of patients in primary care with either asthma (14.8%) or COPD (14.5%), suggesting that asthma may be over-recorded in people with COPD in electronic health records.

In the asthma questionnaire, details were requested on evidence of airway obstruction, current symptoms, smoking history, respiratory comorbidities, and Quality and Outcomes Framework (QOF) indicators [QOF is a national financial incentive scheme for GPs in the UK encouraging regular disease indicator measurement and recording]. The COPD questionnaire requested information on COPD diagnosis, smoking history, symptoms, spirometry, confirmation by a respiratory physician, and respiratory comorbidities. Additional information available from the medical record, including spirometry printouts and letters from respiratory physician or hospitals, was also requested. Data were encrypted twice to ensure anonymity. If a questionnaire was returned blank or every question was answered ‘unknown’, it was considered invalid.

### Database

The CPRD GOLD is a large anonymised UK primary care database that is representative of the UK population with regard to age and sex.^9^ Diagnostic accuracy has been demonstrated to be high in CPRD GOLD for many conditions,^10^ including asthma and COPD. This database contains detailed clinical information on diagnoses, prescriptions, laboratory tests, symptoms, and hospital referrals of included individuals in addition to basic sociodemographic information recorded by the GPs. In the original validation studies, lists of medical codes (Read codes) deemed as specific for asthma or COPD were used to select algorithms to identify patients with asthma and patients with COPD; these codes have a high validity in their respective validation studies. Read codes are part of a hierarchical clinical coding system that is used in GP practices in the UK; each Read code is linked to a specific string of text, which refers to a single diagnosis or symptom.

### Primary outcome and measurements

The primary outcome for this study was the proportion of patients with either asthma or COPD who had the other disease in the validated asthma and COPD populations. The presence of a diagnostic asthma Read code and positive reversibility tests supported an asthma diagnosis in the COPD population. The presence of a diagnostic COPD Read code, smoking history, and fixed airflow obstruction supported a current COPD diagnosis in the population with validated asthma. Spirometry measurements with at least one airflow measurement with forced expiratory volume in 1 second to forced vital capacity ratio (FEV1/FCV) ≤70% were considered as evidence for an obstructive airflow limitation. The quality of the spirometry procedure undertaken in UK primary care to diagnose COPD is high, as determined in a previous validation study.^11^

Possible misdiagnosis and/or lacking diagnosis of asthma in patients with validated COPD, and vice versa, were examined using spirometry measurements, results of reversibility tests, and smoking history. To study the temporality of recorded diagnostic Read codes in patients with concomitantly recorded asthma and COPD, the researchers reported the proportion of patients where the time lapse between the date of validation of one disease and the last known diagnosis of the other disease was >2 years. This methodology was adopted when the researchers learned, from the validation studies, that a patient with COPD would sometimes receive their first asthma diagnosis in the 2 years leading up to the first COPD diagnosis. An asthma code shortly before a first diagnosis of COPD is likely to be a misdiagnosis of asthma. If the asthma code was given multiple years before the COPD diagnosis, asthma before COPD onset is more probable.

Conversely, if the last COPD code was given >2 years before the validation of an asthma diagnosis (and the validated asthma diagnosis is assumed to be true), the COPD might be misdiagnosed as the code was not repeated afterwards.

Asthma and COPD diagnoses are based on symptoms, signs, and spirometry, but there is no clear reference test. A panel consisting of two physicians determined whether asthma or COPD were present in the validated patients using all available information, and according to national and international guidelines. Both physicians were blind to the patient selection algorithm and adjudicated the asthma and COPD statuses independently. Where opinion differed, the cases were discussed and agreement was reached by consensus.

### Statistical analysis

The researchers calculated the proportion of patients with asthma with COPD and vice versa with 95% confidence intervals (CIs) using exact binomial Clopper–Pearson intervals. Cells with less than five entries were merged for presentation. All analyses were conducted using Stata (version 14.0) in 2017.

## RESULTS

### Background characteristics

The baseline characteristics of the 751 patients with confirmed asthma and COPD diagnoses are shown in Table 1. Among patients with validated asthma, those with a COPD diagnosis were older than those without (67 and 45 years, respectively). There was no noticeable difference in mean age between patients with validated COPD with or without an asthma Read code (73 years in both groups). Most of the validated asthma study population was female (61%), whereas the validated COPD population was more evenly split regarding sex (51% male). Table 1 is further split into two age categories: in the patients with validated asthma, a concomitant COPD diagnosis is more likely when the patient is >50 years of age. Only a small percentage of validated patients with COPD were ≤50 years of age.

**Table 1.** Baseline characteristics of patients with confirmed asthma and COPD diagnoses (*N* = 751)

**Characteristic**	**Asthma validation**			**COPD validation**		
	
	**COPD Read code**	**No COPD Read code**	**Total**	**Asthma Read code**	**No asthma Read code**	**Total**
**All patients**						
Individuals, *n* (%)	52 (15)	299 (85)	351	210 (52)	190 (48)	400
Mean age, years (95% CI)	67 (64–70)	45 (42–47)	48 (46–50)	73 (71–74)	73 (72–75)	73 (72–74)
Sex, male, *n* (%)	22 (42)	114 (38)	136 (39)	99 (47)	104 (55)	203 (51)
(Ex-)smoker,^a^ *n* (%)	43 (82)	112 (37)	155 (44)	–	–	–

**Patients aged** ≤**50 years**						
Individuals, *n* (%)	4 (2)	172 (98)	176	9 (75)	3 (15)	12
Sex, *n* male (%)	2 (50)	73 (42)	75 (43)	2 (22)	2 (67)	4 (33)
(Ex-)smoker^a^, *n* (%)	4 (100)	48 (28)	52 (30)	–	–	–

**Patients aged** >**50 years**						
Individuals, *n* (%)	48 (27)	127 (73)	175	201 (52)	187 (48)	388
Sex, male, *n* (%)	20 (42)	41 (32)	61 (35)	97 (48)	102 (55)	199 (51)
(Ex-)smoker^a^, *n* (%)	39 (81)	64 (50)	103 (59)	–	–	–

a COPD population was preselected to only include (ex-)smokers. COPD = chronic obstructive pulmonary disease.

### Patients with validated asthma

In total, 351 patients with a validated asthma diagnosis were studied, of whom 52 (15%) had a recorded COPD Read code. Details are summarised in Figure 1. For six of the 52 patients with asthma and with COPD codes, the COPD codes were given >2 years prior to asthma validation. For the remaining 46, COPD codes were within 2 years of the asthma validation date. Of the 46 with validated asthma and recent COPD codes, 38 were smokers or ex-smokers and eight were recorded as never-smokers. Out of 299 patients with asthma without COPD codes, 112 were (ex-)smokers, whereas 187 were recorded as never-smokers.

Concomitant asthma and COPD in patients with validated asthma was assumed in the following scenarios: if the patients with validated asthma had a recent diagnosis of COPD and were smokers or (ex-)smokers (*n* = 38); or if they showed obstruction on their spirometry and were smokers or (ex-)smokers but lacked a COPD code (*n* = 14). As such, concomitant asthma and COPD was likely in 52 patients (14.8%, 95% CI = 11.3 to 19.0)

Solitary asthma (without COPD) was assumed in patients with validated asthma in three scenarios: either if they did not have a COPD code or showed obstruction on lung function tests (*n* = 187 never-smokers and *n* = 98 ex-smoke); if they had a past COPD code >2 years ago (as the coding should have been repeated) (*n* = 6); or if they had a recent COPD code but no smoking history (*n* = 8). As such, a solitary diagnosis of asthma was likely in 299 patients (85.2%, 95% CI = 81.0 to 88.7).

### Patients with validated COPD

A total of 400 patients with a validated COPD diagnosis were studied, of which 210 (52.5%) had a recorded asthma Read code. Details are summarised in Figure 2. For 82 of the 210 patients with COPD with asthma codes, the asthma codes were >2 years prior to COPD validation. For the remaining 128, asthma codes were within 2 years of the COPD validation date. Of the 128 with validated COPD and recent asthma codes, 42 had a recording of positive reversibility testing and 86 did not have a recording of positive reversibility testing. Out of 190 patients with COPD without asthma codes, 16 had a recording of positive reversibility testing, whereas 174 did not have lung function tests indicating reversibility of their airflow obstruction.

Concomitant asthma and COPD were assumed in validated patients with COPD in two scenarios: validated patients with COPD and with a recently recorded asthma code and a recording of positive reversibility testing (*n* = 42); and validated patients with COPD and without a recent asthma code but with positive reversibility testing recorded (*n* = 16). As such, concomitant asthma and COPD was likely in 58 patients (14.5%, 95% CI = 11.2 to 18.3).

Solitary COPD (without asthma) was assumed in patients with validated COPD based on the following criteria: validated patients with COPD with no asthma codes or recording of positive reversibility testing (*n* = 174); validated patients with COPD where the last asthma code was >2 years before asthma validation (indicating asthma prior to COPD) (*n* = 82); and validated patients with COPD with recent asthma codes but without positive reversibility testing (*n* = 86). As such, COPD without clear evidence of current asthma was likely in the remaining 342 patients (85.5%, 95% CI = 81.7 to 88.8).

## DISCUSSION

### Summary

The researchers investigated the prevalence of COPD in patients with validated asthma and vice versa using CPRD GOLD data on smoking, spirometry, and reversibility testing; in addition to detailed GP questionnaires and supporting information including outpatient referral letters, other emergency department discharge letters, airflow measurements, and radiography records. The main finding of this study was that the 14.8% of patients with validated asthma had a concurrent COPD diagnosis, whereas 14.5% of validated patients with COPD had a concurrent asthma diagnosis. Asthma may also be over-recorded in CPRD GOLD for patients with COPD.

However, more than half of the patients with validated COPD had previously received an asthma diagnosis Read code, suggesting that overdiagnosis of asthma in patients with COPD commonly occurs, particularly early in the diagnostic process. Overdiagnosis of COPD in patients with asthma is less likely.

### Strengths and limitations

This study has potential limitations that need consideration. First, the results of this study are only applicable to the records in the CPRD, although this database is considered representative of the general UK population.^9^

Second, only patients with asthma and patients with COPD for whom their GP responded to verify their diagnosis in the original questionnaire were included in this study. GPs of more complicated cases might be less likely to respond where diagnostic uncertainty may exist. However, this issue is mitigated to an extent as GPs were paid for providing the information for validation, and the baseline characteristics of the individuals for whom a questionnaire was returned were similar to the characteristics for which no questionnaire was returned.^8^ Data on eligible patients who were not included, as there was no returned questionnaire, were available from CPRD GOLD, but did not contain all the information of a completed questionnaire.

Third, the validation process was mostly based on the GP questionnaires, which are available in the original studies.^8^ Additional information (discharge letters, spirometry measurements, and radiography) were available for 31.5% of patients with asthma. This means the strength of evidence for confirmation or rejection of recorded diagnosis in CPRD varied among the participants, and a panel diagnosis for chronic respiratory diseases can be considered as subjective. In the original validation studies, positive predictive values were calculated separately for people for whom additional information was and was not provided, with similar results in these sensitivity analyses.

Fourth, the researchers assumed the samples were representative of the asthma and COPD populations, whereas both sampling methods were based on possible identification strategies. The identification strategies or algorithms used for sampling are described in detail in their respective validation studies.^8^^,^^12^

Fifth, this study clarifies the burden of concomitant asthma and COPD diagnosis in primary care, but additional information on how these patients are treated is needed in further studies.

Sixth, the COPD population was selected to include only current or ex-smokers. This implies that this study’s findings in the COPD populations are only valid for (ex-)smokers.

Finally, GPs could have more information on the clinical status, which was not shared in the questionnaire. This risk is present but diminished as the provision of additional information was remunerated.

This study has a few strengths. First, it was possible to quantify the burden of concomitant diagnosis of asthma and COPD in a large cohort of primary care patients (CPRD GOLD), which is representative in terms of age and sex to the general UK population. Second, information included in both the CPRD GOLD and in the questionnaires sent out for the original validation studies was used in order to differentiate between asthma and COPD. Finally, this study adds to the relatively small body of literature on the epidemiology of concomitant asthma and COPD in primary care.

### Comparison with existing literature

In primary care, most consultations on respiratory diseases start with a provisional diagnosis made on clinical grounds from the patients’ symptoms, in addition to previous specialists’ correspondence if available.^13^^–^16 Spirometry is needed to accurately differentiate the diagnosis of asthma and COPD, but is not always used in a primary care setting.^17^^–^20

The prevalence of asthma in COPD populations is lower compared with many previously published studies, especially those based on electronic health records. However, the prevalence of COPD in asthma populations is similar to a previous cross-sectional study measuring the prevalence for comorbidities in asthma, which reported that 13.4% of patients with asthma had a COPD diagnosis compared with 3.4% of the remaining general population.^21^ A previous systematic review stated a pooled prevalence of asthma in patients with COPD of 27%, with considerable heterogeneity^12^ The definition of asthma and COPD diagnosis tends to differ between studies, which might explain this observation. A study using Norwegian GP data confirmed COPD diagnosis using spirometry in 17.1% of patients with only a previous asthma diagnosis.^22^ Among subjects with a spirometry-based study diagnosis of COPD in GP practices in Scotland and the US, 51.5% reported a prior diagnosis of asthma without a concurrent chronic bronchitis or emphysema diagnosis.^23^ A systematic review on ACOS in 2015 found a pooled prevalence of 27% in population-based studies of patients with COPD and 28% in hospital-based studies of patients with COPD.^12^ A recent multicentre study on patients with COPD in Japan found an asthma prevalence of 9.2% or 4.2%, depending on the FEV1 cut-off.^24^ Other studies report a very wide range of prevalence of concomitant asthma and COPD as the diagnosis criteria are heterogeneous and a consensus on diagnostic criteria is needed.^4^^,^^5^

### Implications for research and practice

This study suggests that overdiagnosis of asthma in patients with COPD is more likely than overdiagnosis of COPD in patients with asthma. COPD is possibly more conservatively diagnosed as it is considered a more severe disease, whereas asthma can be more liberally diagnosed. In addition, a patient with COPD can be diagnosed with asthma in the years before first COPD diagnosis, after which no further recording of asthma is made, suggesting that the asthma diagnosis was likely to be false. In patients with presumed concomitant diagnosis of asthma and COPD, reversibility testing can be used to verify the asthma diagnosis.

The findings from the present study have implications for further research into concomitant asthma and COPD. Identifying potential concomitant asthma and COPD using electronic health records should be done cautiously. If only a single code for both diseases is required for the identification algorithm, the prevalence of concomitant diagnosis of asthma and COPD is likely to be overestimated.

In addition, this study also has implications for the management of patients with COPD with a past asthma diagnosis, as the previous asthma diagnosis might be either outdated or misdiagnosed. Incorrect management can expose patients to adverse effects and incur additional costs for them and the health system; for example, through unnecessary medication regimens such as the usage of montelukast in patients with COPD. This study did not go into detail on the current treatment of either patients with validated asthma or patients with validated COPD.
